# Clozapine Patients at the Interface between Primary and Secondary Care

**DOI:** 10.3390/pharmacy6010019

**Published:** 2018-02-26

**Authors:** Marita Barrett, Anna Keating, Deirdre Lynch, Geraldine Scanlon, Mary Kigathi, Fidelma Corcoran, Laura J. Sahm

**Affiliations:** 1 Pharmacy Department, Cork University Hospital, Cork T12 DC4A, Ireland; anna.keating@hse.ie (A.K.); deirdrem.lynch@hse.ie (D.L.); 2 Adult Mental Health Unit, Cork University Hospital, Cork T12 DC4A, Ireland; geraldine.scanlon@hse.ie (G.S.); mary.kigathi@hse.ie (M.K.); fidelma.corcoran@hse.ie (F.C.); 3 School of Pharmacy, University College Cork, Cork T12 YN60, Ireland; l.sahm@ucc.ie; 4 Pharmacy Department, Mercy University Hospital, Cork T12 WE28, Ireland

**Keywords:** Clozapine, Pharmacy, drug-drug interactions, patient safety

## Abstract

Patients receiving clozapine must undergo routine blood monitoring to screen for neutropenia, and to monitor for potential agranulocytosis. In Cork University Hospital, Cork, Ireland, clozapine is dispensed in the hospital pharmacy and the pharmacists are not aware of co-prescribed medicines, potentially impacting upon patient safety. The aim of this study was to examine the continuity of care of patients prescribed clozapine. A retrospective audit was conducted on patients attending the clozapine clinic at Cork University Hospital and assessed patients’ (i) independent living, (ii) co-prescribed medicines and (iii) knowledge of their community pharmacists regarding co-prescribed clozapine. A list of prescribed medicines for each patient was obtained, and potential drug-drug interactions between these medicines and clozapine were examined using Lexicomp^®^ and Stockley’s Interaction checker. Secondary outcomes included patients’ physical health characteristics, and a review of co-morbidities. Data were collected between the 29 May 2017 and 20 June 2017. Local ethics committee approval was granted. Patients were eligible for inclusion if they were receiving clozapine treatment as part of a registered programme, were aged 18 years or more, and had the capacity to provide written informed consent. Microsoft Excel was used for data analysis. Of 112 patients, (33% female; mean age (SD) 43.9 (11.3) years; 87.5% living independently/in the family home) 86.6% patients reported that they were taking other prescribed medicines from community pharmacies. The mean (SD) number of co-prescribed medicines in addition to clozapine was 4.8 (4) per patient. Two thirds of community pharmacists were unaware of co-prescribed clozapine. Interactions with clozapine were present in all but 3 patients on co-prescribed medicines (*n* = 97). Lexicomp^®^ reported 2.9 drug-drug interactions/patient and Stockley’s Interaction Checker reported 2.5 drug-drug interactions/patient. Secondary outcomes for patients included BMI, total cholesterol, and HbA_1c_ levels, which were elevated in 75%, 54% and 17% respectively. Patients prescribed clozapine did not receive a seamless service, between primary and secondary care settings. Community pharmacists were not informed of clozapine, prescribed for their patients, in two thirds of cases. Patients in this study were exposed to clozapine-related drug-drug interactions and hence potential adverse effects. This study supports reports in the literature of substandard management of the physical health of this patient group. This study shows that there is an opportunity for pharmacists to develop active roles in the management of all clozapine-related effects, in addition to their traditional obligatory role in haematological monitoring. This study supports the need for a clinical pharmacist to review inpatients commencing on clozapine, monitor for drug-drug interactions and provide counselling.

## 1. Introduction

Clozapine is the medication of choice for Treatment Resistant Schizophrenia; as its efficacy is superior to other antipsychotic agents [1]. Despite the benefits of clozapine therapy, it is underutilised due to safety concerns and strict monitoring requirements. Patients prescribed clozapine must have their bloods monitored routinely to screen for neutropenia, and to monitor for potential agranulocytosis. Monitoring is carried out in secondary care in some countries including Ireland, often from designated clozapine clinics. Hospital pharmacists supply clozapine only after obtaining a valid prescription and blood result. 

The life expectancy of people with schizophrenia is 20% lower than the general population. Men with this diagnosis die, on average, 20 years earlier than those without schizophrenia [2]. Most of the increased mortality is due to higher levels of cardiovascular disease (CVD) and physical health problems [2]. The incidence of obesity, type 2 diabetes mellitus (T2DM) and hyperlipidaemia is higher than in the general population. Hyperlipidaemia is up to five times higher in those prescribed antipsychotics than in those who are not, and over half of these have low High Density Lipoprotein Cholesterol (HDL-C) and raised triglycerides [3]. This increases the risk of developing metabolic syndrome. Unemployment, tobacco use and alcohol misuse contribute to this increased CVD risk, in addition to the metabolic effects of antipsychotic agents; in particular clozapine [2,3]. Patients prescribed clozapine, with co-morbidities, whether clozapine-induced or not, will also be prescribed and taking other medications. These are prescribed by General Practitioners (GPs) and dispensed by community pharmacists, thus adding to their burden by attending multiple healthcare professionals (HCPs) at different locations, including both hospital and community pharmacies [4]. There is evidence in the literature about the communication gap that exists between primary and secondary care services for patients prescribed clozapine [5]. Obtaining medicines from multiple sources leads to fragmented service delivery, compromises safe use of medicines and, exposes patients to an increased risk of drug-drug interactions (DDIs) and adverse effects [5,6]. There is little published on the consequences of disjointed pharmacy services for those patients who receive clozapine separate to their other medicines. Additionally, these patients may be at risk of the sequelae of poor communication between physicians in primary and secondary care services [7]. Consequently, errors may occur when patients transition between these services or there may be substandard management of clozapine-associated physical comorbidities as a result of ambiguity, between prescribers, as to where responsibility lies [2,8,9].

Many of clozapine’s pharmacodynamic and pharmacokinetic interactions are reported in the literature [1,5]. Community pharmacists however, are unable to monitor for clozapine-related adverse effects or provide appropriate counselling to the patient, if they are unaware that it has been prescribed [5]. Polypharmacy (the prescribing of five or more medications) is increasing in patients with schizophrenia, another factor that contributes to DDIs and adverse drug events [10].

There are other models that manage clozapine therapy differently: In New Zealand and Australia, clozapine is now available from community pharmacies [4]. One benefit is that community pharmacists know all co-prescribed medicines, and can therefore monitor for interactions and counsel the patient appropriately [4]. Knowledge of co-prescribed clozapine means they are ideally positioned to monitor for associated side effects, provide advice on the management of these, and recommend alleviating treatments [4]. Whether clozapine is dispensed by hospital or community pharmacists, the literature agrees that patients prescribed clozapine should be provided with an integrated holistic pharmacy service [4,8,11,12].

In Cork University Hospital (CUH), Ireland, patients who require clozapine therapy are commenced as an inpatient in the Acute Mental Health Unit (AMHU) of the hospital. As the AMHU does not have a clinical pharmacist, patients’ medicines are not reviewed by a clinical pharmacist prior to initiation. Once discharged, these patients receive clozapine from CUH pharmacy department, in the absence of any information regarding co-morbidities or concomitant medications. There is no formal system in place to inform community pharmacists of clozapine therapy. The aim of this study was to describe the impact of non-integration of pharmacy services, upon patient care. 

This study examined:

- The prevalence of patients prescribed clozapine who were living in a community setting; their demographics, physical health characteristics, co-morbidities and co-medications.
- Community pharmacists’ awareness of clozapine prescription for their patients as recorded in their Patient Medication Record (PMR). This is the unique record for each patient held by community pharmacies on their computer systems.
- Co-prescribed medicines in this cohort.
- DDIs between clozapine and co-prescribed medicines.

## 2. Methods

Permission for conducting this research study was granted by both, the Clinical Research and Ethics Committee (CREC) of the Cork Teaching Hospitals, and the Quality Department of CUH. All data were saved on a password-encrypted laptop. Research Setting and Participants: This study was carried out at the Clozapine Clinic, CUH. The Clozapine Clinical Nurse Specialists (CCNS) take routine bloods and records observations for patients prescribed clozapine. At initiation of the study, there were 141 adult patients registered. Patients were given information about the study. Written informed consent was obtained from patients willing to participate. Inclusion criteria: All patients aged 18 years or over, and registered with the Clozaril Patient Monitoring Service (CPMS) were included. Exclusion criteria: those who did not attend the clinic during the study period (29 May–20 June 2017), and those deemed by the CCNS to lack capacity to provide informed consent, were excluded from the study. 

The following data were collected from participants:

- Patient characteristics: gender, weight, height and blood pressure
- Lifestyle factors: smoking status and living arrangements
- Co-prescribed medicines, and the name of the patient’s community pharmacy

After the clinic, the following data were obtained for each study participant:

- Age
- Number of years taking clozapine as recorded on the CPMS website
- The total daily dose of clozapine as per the prescriptions for dispensing in the pharmacy department in CUH
- A list of medicines obtained from the nominated community pharmacy
- Community pharmacists were also asked:
  1. “Are you aware that Mr Y/ Ms X is receiving clozapine?”
  2. If yes, “Has this been documented in the patient’s PMR?”
  3. “Is clozapine listed on prescriptions from the GP, for this patient?”
- For those that were inpatients during the study period (both newly initiated and long stay patients), the list of co-prescribed medicines was obtained from their inpatient medication prescription and administration record.
- An interaction check was undertaken between clozapine and all medicines, using Lexicomp^®^ [13] and Stockley’s Interaction Checker (SIC) [14]. Lexicomp^®^ is a widely accessible drug interaction software program and studies show it provides the most competent, complete, and user-friendly applications [15]. SIC is a comprehensive and authoritative international reference book on DDIs. DDIs were graded as:
  1. Avoid Combination: Contra-Indicated (CI) /Life Threatening
  2. Consider Therapy Modification/Dose Adjustment
  3. Monitor Therapy
- Co-morbidities as documented in the patient’s records sourced from the medical records department.
- Physical health characteristics including lipid profile and glycosylated haemoglobin (HbA_1c_) were sourced from the laboratory within CUH (iLab), where they are measured on an annual basis.

All data obtained from the retrospective audit were entered into Microsoft Excel and descriptive statistics were performed.

## 3. Results

Of a total of 141 patients registered for clozapine therapy, five patients were deemed too unwell by the CCNS to participate, and 19 patients did not attend the clinic during the study period. Of 117 eligible patients, five patients did not agree to participate, leaving 112 patients, who agreed and provided written informed consent. The demographics of the participants are shown in Table 1. Table 2 details the frequency and type of co-morbidities of participants.

Community Pharmacy Awareness

The majority of patients prescribed clozapine reported that they were prescribed additional medicines in primary care (*n* = 97; 86.6%). Of these 97 patients, 89 had a regular community pharmacy that was contacted, the remaining eight patients did not record having a regular pharmacy. Pharmacists were aware of co-prescribed clozapine for thirty of these patients (33.7%), but for only 20, was this recorded in their PMR. The GP had clozapine on prescriptions for ten of these patients. 

Drug-Drug Interactions

Patients (*n* = 97) were taking a total of 535 prescribed medicines in addition to clozapine. The mean number of co-prescribed medicines (not including clozapine) per patient was 4.8 (SD ± 4.) and ranged from zero to 21. 

Medicines for each patient were checked for interactions with clozapine using two separate reference sources; Lexicomp^®^ and SIC. Of those; 60% (322/535) of medicines had documented interactions with clozapine using Lexicomp^®^ and, 51.5% (277/535) using SIC. 

**Figure 1:**
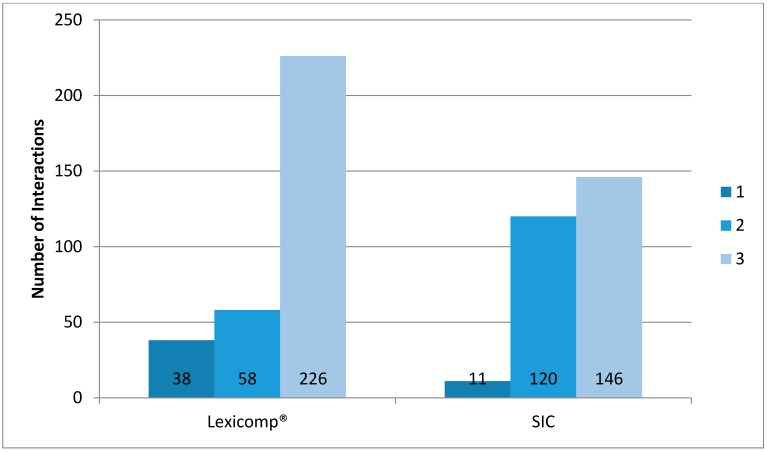
Drug-drug Interactions (DDIs) with Clozapine using Lexicomp and Stockley’s Interaction Checker (SIC) (**1** = *Avoid Combination: Contra-indicated (CI) /life threatening*, **2** = *Consider Therapy Modification/Dose Adjustment*, **3** = *Monitor therapy*).

Figure 1 shows the categories of DDI with clozapine in this study group for both reference sources. 

For those patients taking co-prescribed medicines (*n* = 97); Lexicomp^®^ recorded 3.3 DDIs per patient and SIC recorded 2.9 DDIs per patient.

### 3.1. ‘Avoid Combination: Contra-Indicated (CI) /Life Threatening’ DDIs

The most frequently prescribed contra-indicated (CI) drugs were amisulpride (Lexicomp^®^) and domperidone (SIC) (Table 3). Both domperidone and flupentixol are classified as CI in both references. With regard to amisulpride, SIC does not classify it as CI with clozapine, but does advise that concurrent use might increase the risk of QT prolongation and torsade de pointes (TdP), and that there should be further monitoring in those at risk [14]. 

### 3.2. ‘Consider Therapy Modification/Dose Adjustment’ DDIs

These interactions advise either therapy modification, a dose adjustment or close monitoring. Both Lexicomp^®^ and SIC include benzodiazepines in this category (Table 4). SIC reports several cases describing severe hypotension, respiratory depression, and potentially fatal respiratory arrest in patients taking benzodiazepines and clozapine [14]. Dizziness and sedation are also increased. Lexicomp^®^ also includes hypersalivation, unconsciousness and delirium as risks with concurrent use of clozapine and benzodiazepines [13]. 26.8% (*n* = 30) of patients in this study were taking a benzodiazepine with their clozapine.

SIC grades all anti-hyperglycaemic agents in this category as *“clozapine has been associated with glucose intolerance and therefore might affect diabetic control”* [14]. 

Many selective serotonin reuptake inhibitors (SSRIs) are included in this category, as they increase the risk of QT prolongation/TdP. In addition, sertraline, paroxetine and fluoxetine increase clozapine levels. Mirtazapine can cause additive sedative effects, blood dyscrasias and potentially fatal agranulocytosis with concurrent clozapine therapy [14]. 

Ciprofloxacin increases clozapine concentrations and cases of clozapine toxicity have been reported when taken concomitantly [14]. It also has QT prolonging effects, as have the macrolide antibiotics. 

### 3.3. ‘Monitor therapy’ DDIs

The recommendation for this category of DDIs is to monitor therapy and provide guidance. In this study, 70% of clozapine’s DDIs fall in this category in Lexicomp^®^ and 53% in SIC. 

Medicines causing DDIs with clozapine listed in Lexicomp^®^ include blood pressure lowering agents, antipsychotics, serotonin noradrenaline reuptake inhibitors (SNRIs) and some SSRIs and anticholinergic agents. These can have additive pharmacodynamics effects with clozapine. Other agents include anti-hyperglycaemic agents which may be antagonised by clozapine, and the proton pump inhibitor (PPI) omeprazole which may decrease serum concentration of clozapine. Similar agents are listed in SIC. Of note, SIC lists sodium valproate in this category. Valproate can have a minor effect on clozapine concentrations and advice is provided to monitor for the additive effects of weight gain and central nervous system depressant effects with concomitant clozapine. This is important as almost 22% of the study group were prescribed sodium valproate. Combined hormonal contraception is also in this category, as it can increase clozapine levels. Two patients were taking the oral contraceptive pill (OCP), and hence both will require monitoring if their OCP is discontinued.

Other findings in relation to co-prescribed medicines include 28% of patients were prescribed antiepileptic drugs (AED) or mood stabilizers (MS), 43% were prescribed another antipsychotic and 19% were taking both AED/MS and another antipsychotic in addition to clozapine. Another finding that has implications on clozapine is concomitant nicotine replacement therapy (NRT). Four patients were prescribed NRT, therefore highlighting changes in smoking status. Polycyclic aromatic hydrocarbons in cigarette smoke cause CYP1A2 enzyme induction which reduces clozapine levels. Cessation of smoking can increase plasma clozapine concentrations, thus leading to an increase in adverse effects [16].

#### Medical Complications/Adverse Effects

Body mass index (BMI) was within recommended target for just a quarter of patients (*n* = 26) (Table 5).

The documented prevalence of hypercholesterolemia/dyslipidaemia in the study group was estimated at over 30%. However, when the level of hypercholesterolemia and dyslipidaemia was assessed using the laboratory values recorded, this figure increased to 70%. Dyslipidaemia is defined as elevated total cholesterol (TC) >5 mmol/L OR elevated fasting triglycerides (TGs) >1.7 mmol/L OR elevated fasting low density lipoprotein cholesterol (LDL-C) >3 mmol/L or decreased HDL-C <1 mmol/L for males and <1.3 mmol/L for females [17].

Table 6 shows the breakdown for TC. TC as reported on iLab was elevated for 54% of the study group.

When laboratory TC levels and diagnoses recorded were compared, it was found that some patients with elevated TC levels did not have a diagnosis documented in their medical notes, nor were they prescribed lipid lowering agents. In total 41% (*n* = 46) patients had elevated cholesterol levels on iLab and were undiagnosed/untreated. A total of 36 patients had a documented diagnosis of dyslipidaemia and/or were prescribed lipid lowering agents. Of those, 58% (*n* = 21) were treated to target. Despite a diagnosis of dyslipidaemia and/or taking lipid lowering treatment, 42% (*n* = 15) had elevated TC levels.

The prevalence of T2DM, based on documentation in the medical notes, or associated drug treatment is 19% (*n* = 21). The laboratory marker used by the clozapine clinic for T2DM is HbA_1c_ (Table 7). 

There were nine patients with elevated laboratory HbA_1C_ levels, indicative of pre-diabetes, not prescribed anti-diabetic treatment [20] and a further patient whereby a level >48 mmol/mol was measured but neither diagnosis nor treatment were in place.

In the case of those for whom T2DM was documented in the medical notes and/or patients were prescribed anti-hyperglycaemic agents (*n* = 21); six patients were well controlled with HbA_1c_ < 48 mmol/mol, six had elevated levels between 48–53 mmol/mol and nine had levels >53 mmol/mol. 

Hypertension was documented in the medical notes for sixteen patients. Based on readings taken in the clozapine clinic, eight patients had readings >140/90 mmHg. Three of these patients had documented hypertension, whereas five patients were not diagnosed with hypertension and were not prescribed anti-hypertensive treatment. Constipation; a well-known adverse effect of clozapine, was documented for 21% (*n* = 24) of patients. Five of these patients were not prescribed treatment. 

## 4. Discussion

In this study, the population of patients was a similar size to clozapine-related studies reported in the literature. In addition, this study group had similar characteristics to those in other related studies [2,5,10,21,22,23]. There were more males than females, with a mean age of 44 years, taking a mean dose of 350 (±136) mg of clozapine per day. The average BMI was 30 kg/m^2^ and the percentage of patients smoking was at the lower end of figures reported in the literature at 39%, although this was a self-reported figure. There is evidence showing that in comparison to patients with schizophrenia in general; patients prescribed clozapine have a lower frequency of tobacco use [24]. The majority of patients were stabilised on treatment and residing in the community. All outpatients were self-medicating, including those in supported living arrangements, except for one older patient with Parkinson’s disease, who was living in a nursing home. Nearly 90% of patients reported taking other co-prescribed medicines which are dispensed from community pharmacies. Patients were co-prescribed on average five additional medicines, similar to the figure reported by Murphy et al. [5]. 

### 4.1. Community Pharmacy Awareness

Two-thirds of community pharmacists were unaware of co-prescribed clozapine. This is greater than the figure (40%) reported by Murphy et al. [5] in a ‘shared care’ system in Australia. As expected, this provides evidence that shared care improves the sharing of information in relation to clozapine with community pharmacists. These results showed that most patients did not communicate directly with their community pharmacist about clozapine, or were unaware that they could consult their community pharmacist for healthcare advice. This lack of communication between patients and community pharmacists may have been due to associated mental health stigma as reported in the literature [25,26]. There may be a lack of awareness on the services pharmacists can provide, including checking for interactions or the suitability of co-prescribed medicines. Our results are in agreement with the literature as Bell et al. [8], Heald et al. [10] and Murphy et al. [5] reported a lack of communication with community pharmacists on concomitant clozapine use dispensed from secondary care.

One third of community pharmacists who were aware of concomitant clozapine therapy did not reflect this by documenting clozapine use in the patients’ PMR. This showed ambiguity in their role in relation to clozapine as part of the patients’ PMR. This means when a locum pharmacist, or a pharmacist unfamiliar with the patient, is dispensing medications, they will not have this information available to screen for clozapine-related DDIs and adverse effects. Additionally, when clozapine is not recorded in a patient’s PMR, the software will not include clozapine when alerting the pharmacist to interaction risks. Therefore, a community pharmacist who is aware of concomitant clozapine therapy should document this in the PMR to minimise risk and enhance transfer of information for these patients. More education is required for community pharmacists on how to manage this information and the clinical relevance of this. This was an issue that had to be addressed in Australia when clozapine dispensing transferred from hospital to community pharmacy for stable patients in 2015 [6]. A lack of education and inadequate resources were identified as barriers to implementing this service change.

For more than one in ten patients, the community pharmacist reported seeing clozapine on prescriptions from the patients’ GP. In some cases, pharmacists said clozapine was listed on the prescription [endorsed as “hospital only”]. Pharmacists reported that this was good practice by GPs. GPs also have a role to play in ensuring medicines prescribed in primary care are safe with clozapine. Both Murphy et al. [5] and Parker and Somasunderam [7] showed GPs omitted clozapine from their records in up to 50% of cases; however when GPs were more involved in clozapine services this figure reduces to 5%.

### 4.2. Drug-Drug Interactions

‘Stockley’s Drug Interactions’ is a commonly used reference in Ireland and in the UK. Lexicomp^®^ is a US-based resource. In practice SIC would be used first-line in CUH. The categorised severity of an interaction may differ between the grading systems in both sources, but the underlying mechanisms are the same. For patients co-prescribed other medicines (*n* = 97), DDIs with clozapine occurred in all but three patients. This was in keeping with the figure reported by Leung et al. [1]; in a similar sized population DDIs were found in all but 10 patients. There was an average of 2.9 DDI/patient. Guo et al. [27] reported that nearly a quarter of patients with schizophrenia prescribed an antipsychotic were exposed to a major or moderate DDI. Guo et al.’s [27] figures are not specific to clozapine and include all antipsychotics. In this study, which specifically focused on clozapine, the figures were much higher, with 53.5% of patients exposed to a major or moderate interaction as per Lexicomp® and 58% exposure as per Stockley (category 1&2) [13,14].

Guo et al. [27] reported on medicines that interacted with clozapine and found similar results to this study. The most frequent interacting drugs were SSRIs via CYP 2D6 pathways, phenytoin and carbamazepine via CYP3A4 induction and ciprofloxacin via CYP1A2 mechanisms [27]. Many of the interactions reported in this study group involved a risk of QT prolongation and TdP, additive bone marrow suppression and changes to clozapine serum levels. In a review of clozapine DDIs by Markowitz et al. [28], clinical significance was assessed for each interaction. Interactions between clozapine and benzodiazepines, lithium, some SSRIs, carbamazepine and phenytoin were deemed clinically significant [28]. All of these drugs, deemed by Markowitz et al. to have clinically significant interactions with clozapine, were also prescribed for patients attending the CUH clinic. These patients, who were not reviewed by clinical pharmacists, were also not fully reviewed by community pharmacists as, in at least two thirds of cases, they were uninformed of clozapine as part of a patient’s PMR.

Patients in this cohort, who were living in the community, appeared to be functioning, despite interacting medicines. The clinical relevance of these interactions often becomes more prominent at the point of change i.e., drug initiation, dose change or discontinuation. Pharmacists have the knowledge, expertise and skill mix to process complex interactions and decide upon their clinical relevance at the time of dispensing new/changed medicines. They can recommend an alternative agent and/or provide counselling and rationalise drug therapy. The advice for many clozapine related DDIs is to monitor therapy and give guidance. This was not possible for this patient group without clinical or community pharmacist input. For example, four patients were taking regular domperidone despite multiple warnings on the risk of serious cardiac adverse drug reactions [29]. It is CI for those taking QT-prolonging drugs, i.e., including clozapine [29]. Without knowledge of concomitant clozapine, it was not possible to make any recommendation. One patient was found to have taken a course of the antibiotic ciprofloxacin; this has been reported in the literature to increase clozapine levels by up to 500% [1]. Monitoring of smoking status is assigned to the CCNS. 

### 4.3. Medical Complications/Adverse Effects

Poor monitoring of the physical health of patients with schizophrenia is well documented [2,9]. Even so, there is little evidence of communication of this information to other healthcare professionals, who can act upon abnormal results [2,30,31]. Of the second generation antipsychotics, clozapine has the greatest potential to cause metabolic syndrome, thus predisposing patients to weight gain, hyperlipidaemia and hyperglycaemia [3]. In this study, three quarters of patients were overweight or obese, and 70% had laboratory results indicative of dyslipidaemia. Similarly, Bolton et al. report that 88% of their group had dyslipidaemia [21]. Over 40% of this study group had an elevated TC level and were not prescribed lipid lowering therapy. In Crawford et al.’s study, 80% of patients with dyslipidaemia were left untreated [2]. In contrast to Crawford, monitoring was better in this study group as nearly all patients had the recommended parameters measured [2]. The results demonstrated that the treatment of out-of-range parameters was not adequately managed. The results of this study support recommending measurement of waist circumference and fasting glucose for patients attending the clozapine clinic. This would ensure early prevention/prediction of metabolic syndrome onset. Interestingly, Bolton et al. [21] showed that half of their patients had metabolic syndrome, this is in agreement with other literature [3]. With more than three quarters of patients with an elevated BMI, and indications of poor glucose control with elevated HbA_1c_ levels found for both non-diabetic patients and those with T2DM, monitoring for metabolic syndrome is advised.

A fifth of patients in this study had a diagnosis of T2DM. This is in keeping with other studies with patients prescribed clozapine [21]. HbA_1c_ was >53 mmol/mol in over 40% of those with documented T2DM. This signifies poorly controlled T2DM and adds to an already increased CVD risk burden [20]. HbA_1c_ was elevated in 9% of patients not formally diagnosed with T2DM. There is an opportunity to intervene with dietary and lifestyle advice for these patients before overt T2DM manifests. More needs to be done to improve the management of T2DM in this patient group and when doing so, to incorporate the additive effect of clozapine therapy on diabetes management. Pharmacists can provide individual advice and ensure adherence to medication and blood glucose monitoring, while being cognisant of concomitant clozapine therapy.

Whilst clozapine-induced agranulocytosis is the main fear with treatment, and is the main reason for intensive monitoring, recent evidence from pharmacovigilance databases suggests that GI complications (constipation, intestinal obstruction and paralytic ileus) are the leading cause of clozapine-related deaths [32]. Clozapine-induced constipation ranges from 14% to 60% [23,33]. In this study, over a fifth of the population suffered with constipation (21.4%). This may be under-reported as it is self-managed in many cases, and documentation in the medical notes was not up to date for all patients. Leung et al. [1] reported that gastrointestinal hypo-motility can lead to hospitalisation and death. Community pharmacists play an active role in the pharmaceutical management of bowel problems on a daily basis; therefore, they would be a valuable HCP in the ongoing management of these patients. They are also vendors of codeine-based analgesia (OTC), which can contribute to constipation and hence are in a position to caution against the use of codeine-containing products in this cohort.

It is recognised that more needs to be done to improve the physical health of patients with mental health problems. The National Health Service (NHS) in the UK is committed to merging mental and physical health, and has launched ‘*The mental health workforce plan for England*’ [34]. This follows in the steps of Australia and New Zealand where provision of mental healthcare is community based where possible [12,35], and clozapine therapy is not excluded from this. As stated by Knowles et al., community access arrangements “*provide community pharmacists with an opportunity to support clozapine users within the context of their overall medicine regimen, thus promoting holistic care which is an integral part of patient-centred care*” [35].

Patients need education and counselling, and often clozapine counselling is done early in therapy when the patient is too unwell to retain the information provided [36]. There is evidence to show that interventions involving clinical pharmacist counselling can improve patient knowledge and this empowers patients in the management of their treatment [36].

## 5. Limitations

This is a single site study which limits the generalisability of the findings. We have reported on co-prescribed medicines, however additional medicines, (both those which can be bought without prescriptions and complementary and alternative therapy/products) are not included and these may contribute to additional DDIs. As patients can attend any pharmacy; there is a possibility that patients may have received other medications in pharmacies other than the ones given in the study, which may underestimate the extent of co-prescribing.

## 6. Conclusions

Patients prescribed clozapine are currently not receiving a seamless service, caught as they are, at the interface of primary and secondary care. Community pharmacists were not aware of prescribed clozapine in two thirds of cases. Patients in this study were exposed to clozapine-related drug-drug interactions and hence potential adverse effects. This study supports previous work on the sub-optimal management of the physical health of this patient group. There is international evidence to show clozapine can be safely dispensed from community pharmacies, exclusively, or as part of shared care settings. Ireland could follow this approach and make changes to support community dispensing of clozapine for stable patients. The remuneration model for pharmacy services needs consideration; one that would reimburse pharmacist services, including medication reconciliation and discharge summaries. This would serve as a driver to change the current, non-integrated, clozapine practice. Current pharmacy services provided to patients prescribed clozapine in secondary care need reform. There is an opportunity for pharmacist involvement in the management of all clozapine-related effects, in addition to their traditional role in haematological monitoring. This study supports the need for a clinical pharmacist to review inpatients commenced on clozapine, monitor for DDIs and provide counselling. Hospital pharmacists, with the patient’s permission, could liaise with their colleagues in community pharmacies, to ensure continuity of care once patients are discharged. Resources and educational material for community pharmacists will need to be developed. Policies to provide for this change in practice will also need consideration. These patients are community based and current evidence supports empowering patients to remain in the community, whilst providing more integrated care.

## Figures and Tables

**Table 1:** Study Participant Demographics.

Study Participant Demographics	Variable	*n* =	%
Gender	Male Female	75 37	67 33
Age (years)	Mean (SD) Median	43.9 (11.3) 32	- -
Patient type	Outpatient Inpatient	104 8	92.9 7.1
Daily dose of clozapine (mg)	Mean (SD) Median	350 (±136) 312.5	- -
Duration prescribed clozapine (years)	<0.5 ≥0.5–1 >1–5 >5–10 >10	10 4 20 23 55	8.9 3.6 17.9 20.5 49.1
Clozapine Indication	TRS * Psychotic disorder in PD ^~^ Not specified	108 2 2	96.4 1.8 1.8
Smoking status	Smoker Non-smoker	44 68	39 61
Ethnic Origin (as per CPMS)	Caucasian Asian	111 1	99.1 0.9
Living Arrangements	Independent /Family home Other (NH, H) ^#^	98 14	87.5 12.5

* TRS = treatment resistant schizophrenia, ~ PD = Parkinson’s disease, # NH = Nursing Home, H = Hostel, supported accommodation, CPMS = clozaril patient monitoring service, SD = standard deviation, - = not applicable

**Table 2:** Frequency and type of co-morbidities of participants (*n* = 112).

Co-Morbidities of Participants	*n* = *	%
Hypercholesterolemia/dyslipidaemia	35	31.3
T2DM ^#^	21	18.8
Hypothyroidism	12	10.7
Hypertension	16	14.3
Gastrointestinal	43	38.4
(Constipation)	(13)	(30.2)
(GORD ^~^/dyspepsia)	(19)	(44.2)
(Both)	(11)	(25.6)
Respiratory (Asthma & COPD ^≈^)	15	13.4
Cardiovascular disease	9	8
Tachycardia	8	7.1
Known QT prolongation	2	1.8

* patients may have multiple co-morbidities. # T2DM = Type 2 Diabetes Mellitus, ~ GORD = gastro oesophageal reflux disease, ≈ COPD = chronic obstructive pulmonary disease.

**Table 3:** Co-prescribed Contra-indicated (CI)/life-threatening medicines with clozapine.

	Contra-Indicated (CI)/Life-Threatening DDIs	
**Medicine**	Lexicomp (*n* = 38)	SIC (*n* = 11)
Amisulpride	16	-
Citalopram	7	-
Domperidone	4	4
Olanzapine	-	3
Fluoxetine	3	-
Quetiapine	2	-
Inhaled Antimuscarinics	3	-
Fluphenazine	-	2
Trimethoprim	-	1
Carbamazepine	1	-
Flupentixol	1	1
Phenytoin	1	-

**Table 4:** Consider Therapy Modification/Dose Adjustment DDIs with Clozapine.

	Consider Therapy Modification/Dose Adjustment	
	Lexicomp (*n* = 58)	SIC (*n* = 120)
Anti-hyperglycaemic agents	-	34
Benzodiazepines Zolpidem	32 1	32 -
SSRIs Mirtazapine	3 -	19 4
Anti-Epileptic Drugs/Mood Stabilisers -*Lithium* *-Phenytoin* *-Carbamazepine*	- - -	8 1 1
Antipsychotics -*Haloperidol* *-Chlorpromazine*	7 2	7 -
Antibiotics -*Macrolides* *-Ciprofloxacin*	4 1	- 1
Anti-Parkinson’s medicines -*Carbidopa/levodopa* *-Ropinirole*	2 1	2 1
Analgesia -*Codeine* *-Tramadol*	3 2	- -
Omeprazole	-	8
Desmopressin	-	1
Memantine	-	1

**Table 5:** BMI (kg/m^2^).

BMI	(*n* = 112)
≤25	26
26–29	39
30–35	26
>35	19
Not done	2
Mean	29.8 (±6.16)

**Table 6:** Total Cholesterol (TC) Levels (mmol/L) of Participants.

TC (mmol/L)	*n* = 112	%
≤5	47	42
5.1–6	35	31
>6	26	23
Not done	4	4
Mean (SD)	5.3 (1.04)	-

**Table 7:** HbA1c Values for the study participants.

HbA_1c_ (mmol/mol)	*n* = 112	%
≤42 *	79	71
43–47	12	11
48–53 *	7	6
>53	9	8
Not done	5	4
Mean (SD)	40 (±10.7)	

* Levels < 42 mmol/mol are normal [18]. Those >48 mmol/mol confirm T2DM [18,19].
